# Supplementary material for: Variable Temperature Behaviour of the Hybrid Double Perovskite MA2KBiCl6
Source: Molecules. 2022 Dec 25;28(1):174. doi: 10.3390/molecules28010174 (PMC9822144; doi:10.3390/molecules28010174)
Supplement: Supplementary file 1 [file molecules-28-00174-s001.zip › molecules-2117829-supplementary.pdf]

# Variable Temperature Behaviour of the Hybrid Double Perovskite $MA_2KBiCl_6$

**Fengxia Wei [1,*], Yue Wu [2], Shijing Sun [3], Zeyu Deng [4], Li Tian Chew [1], Baisong Cheng [1], Cheng Cheh Tan [1], Timothy J. White [5] and Anthony K. Cheetham [4,6]**

1 Institute of Materials Research and Engineering, Agency for Science, Technology and Research, 2 Fusionopolis Way, Singapore 138634, Singapore
2 Stanford Synchrotron Radiation Lightsource (SSRL), SLAC National Accelerator Laboratory, Menlo Park, CA 94025, USA;
3 Energy and Materials Division, Toyota Research Institute, California, CA 94022, USA
4 Department of Materials Science and Engineering, National University of Singapore, Singapore 117575, Singapore
5 School of Materials Science and Engineering, Nanyang Technological University, 50 Nanyang Avenue, Singapore 639798, Singapore
6 Materials Research Laboratory, University of California, Santa Barbara, CA 93106, USA
* Correspondence: wei_fengxia@imre.a-star.edu.sg

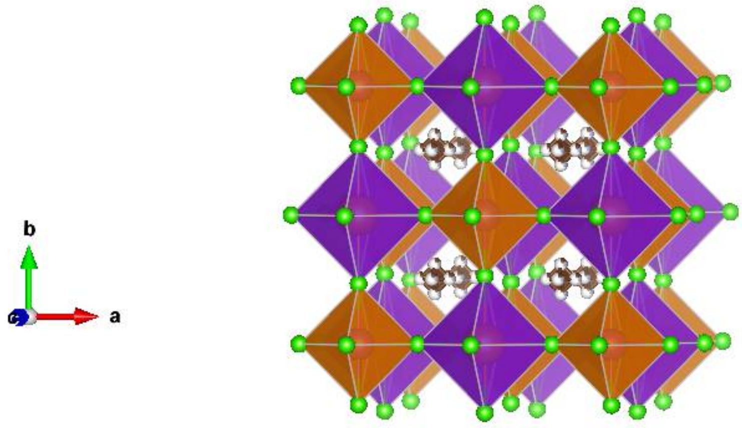


**Figure S1.** The crystal structure of $MA_2KBiCl_6$ double perovskite at 380 K. $BiCl_6$: purple, $KCl_6$: brown. MA cation is disordered in the cavity, H is omitted for illustration purpose.

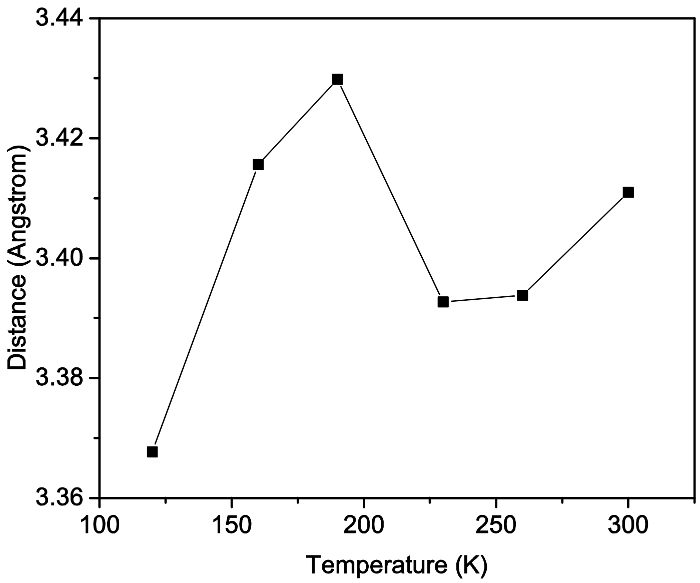

**Figure S2.** Distance between N-Cl of the hydrogen bond N-H···Cl as a function of temperature.

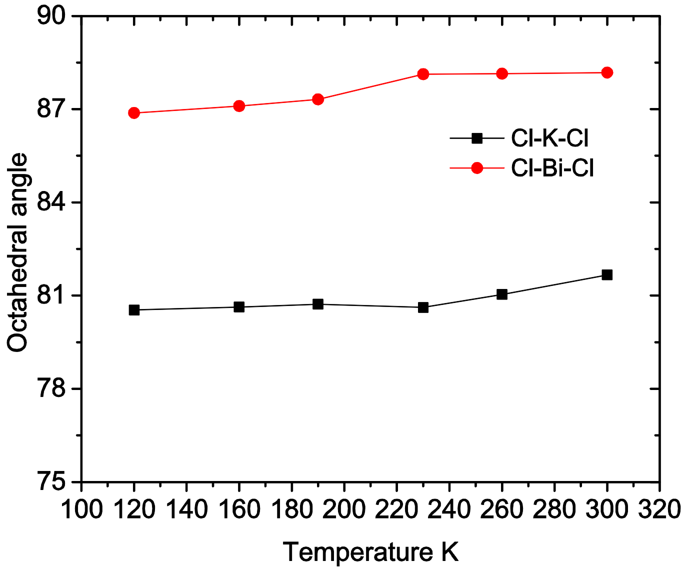


**Figure S3.** Octahedral angles variation with respect to temperature. Only one set of angles for each octahedra are shown (the total angle = 180° when plus the other set of angles). Smaller angle away from 90° means more distortion.

**Table S1.** Atomic coordinates and ADPs at 380K.

| Atom | Wyckoff position | x | y | z | Beq |
|---|---|---|---|---|---|
| Bi | 48d | 0 | 0.5 | 0.5 | 0.0514(8) |
| K | 48d | 0.5 | 0.5 | 0.5 | 0.063(3) |
| Cl | 8d | 0.2357(7) | 0.5 | 0.5 | 0.125(3) |

| | $U_{11}$ | $U_{22}$ | $U_{33}$ |
|---|---|---|---|
| Bi | 0.0514(8) | 0.0514(8) | 0.0514(8) |
| K | 0.063(3) | 0.063(3) | 0.063(3) |
| Cl | 0.055(4) | 0.161(6) | 0.161(6) |

**Table S2.** Experimental details for the HT phase (refinement using OLEX2).

| | |
|---|---|
| **Composition** | $C_2N_2H_{12}KBiCl_6$ |
| **Chemical formula weight** | 472.78 |
| **Temperature** | 380.00(14) K |
| **space group** | $Fm\bar{3}m$ |
| **a (Å)** | 11.4326(2) |
| **Volume (Å$^3$)** | 1494.30(9) |
| **Z** | 4 |
| **mu** | 13.095 |
| **Absorpt correction Tmax** | 0.986 |
| **Absorpt correction Tmin** | 0.981 |
| **Absorpt correction type** | analytical |
| **Crystal colour primary** | colourless |
| **Experimental crystal density diffractions** | 2.048 |
| **Experimental crystal F000** | 816 |
| **Crystal size (mm)** | 0.1417 × 0.1009 × 0.0935 |
| **h** | $-14 < h < 15$ |
| **k** | $-15 < k < 14$ |
| **l** | $-14 < l < 14$ |
| **Diffraction reflections number** | 4380 |
| **Diffraction reflections theta full** | 25.242 |
| **Diffraction reflections theta max** | 27.925 |
| **Diffraction reflections theta min** | 3.086 |
| **Diffraction detector** | CCD plate |
| **Diffraction detector area resol mean** | 16.1183 |
| **Diffraction measurement device** | four-circle diffractometer |
| **Diffraction measurement device type** | Xcalibur, Eos, Gemini ultra |
| **Diffraction measurement method** | \w scans |
| **Diffraction radiation monochromator** | graphite |
| **Diffraction radiation type** | MoK\a |
| **Diffraction radiation wavelength Å** | 0.71073 |
| **Reflections number gt** | 121 |
| **Reflections number total** | 125 |

| | |
|---|---|
| **Reflections completeness** | 100 |
| **Reflections threshold expression** | I > 2\s(I) |
| **Refine Diffraction density max** | 1.846 |
| **Refine Diffraction density min** | −0.817 |
| **Refine Diffraction density rms** | 0.286 |
| **Refine ls extinction coefficient** | 0.0010(5) |
| **Refine ls extinction expression** | $Fc^{*} = kFc[1 + 0.001 \times Fc^{2}\backslash I^{3}/\sin(2\backslash q)]^{-1/4}$ |
| **Refine ls extinction method** | SHELXL-2014/7 |
| **Refine ls goodness of fit ref** | 1.433 |
| **Refine ls number parameters** | 7 |
| **Refine ls number Reflections** | 125 |
| **Refine ls number restraints** | 0 |
| **Refine ls R factor all** | 0.0471 |
| **Refine ls R factor gt** | 0.0458 |
| **Refine ls restrained S all** | 1.433 |
| **Refine ls shift/su max** | 0 |
| **Refine ls shift/su mean** | 0 |
| **Refine ls structure factor coef** | Fsqd |
| **Refine ls weighting details** | $W = 1/[\backslash s^{2}(Fo^{2}) + (0.0794P)^{2}]$ where $P = (Fo^{2} + 2Fc^{2})/3$ |
| **Refine ls weighting scheme** | calc |
| **Refine ls wR factor gt** | 0.1287 |
| **Refine ls wR factor ref** | 0.129 |

**Table S3.** Bond lengths (Å) and angles (°) at different temperatures.

| | 300 K | 260 K | 230 K | 190 K | 160 K | 120 K |
|---|---|---|---|---|---|---|
| **C-N** | 1.327(18) | 1.36(18) | 1.386(3) | 1.434(2) | 1.455(2) | 1.472(3) |
| **K-Cl** | 3.048(13) | 3.046(14) | 3.042(2) | 3.072(2) | 3.078(2) | 3.081(3) |
| **Bi-Cl** | 2.678(13) | 2.675(14) | 2.671(2) | 2.694(2) | 2.697(2) | 2.699(3) |
| **K-Cl-Bi** | 172.998(8) | 172.553(8) | 172.261(12) | 161.054(12) | 159.413(11) | 157.979(12) |

**Table S4.** Experimental details for the LT phase, refinement using JANA.

| | |
|---|---|
| **Composition** | $C_2N_2H_{12}KBiCl_6$ |
| **Chemical formula weight** | 472.78 |
| **Temperature** | 180.00(14) K |
| **space group** | $R\bar{3}m$ |
| **a (Å)** | 7.7379(7) |
| **c (Å)** | 21.1415(16) |
| **Volume (Å$^3$)** | 1096.26(16) |
| **Z** | 3 |
| **mu** | 13.371 |
| **Absorpt correction Tmax** | 0.542 |
| **Absorpt correction Tmin** | 0.292 |

| | |
|---|---|
| **Absorpt correction type** | analytical |
| **Crystal colour primary** | colourless |
| **Experimental crystal density diffractions** | 2.3297 |
| **Experimental crystal F000** | 690 |
| **Crystal size (mm)** | 0.182 × 0.134 × 0.082 |
| **h** | $-5 < h < 9$ |
| **k** | $-4 < k < 10$ |
| **l** | $-24 < l < 26$ |
| **Diffraction reflections number** | 1493 |
| **Diffraction reflections theta full** | 27.34 |
| **Diffraction reflections theta max** | 27.96 |
| **Diffraction reflections theta min** | 2.89 |
| **Reflections number gt** | 340 |
| **Reflections number total** | 340 |
| **Reflections completeness** | 100 |
| **Reflections threshold expression** | I > 3\s(I) |
| **Refine Diffraction density max** | 1.42 |
| **Refine Diffraction density min** | −1.99 |
| **Refine ls extinction method** | none |
| **Refine ls goodness of fit ref** | 2.02 |
| **Refine ls number parameters** | 20 |
| **Refine ls number Reflections** | 340 |
| **Refine ls number restraints** | 0 |
| **Refine ls R factor all** | 0.0321 |
| **Refine ls R factor gt** | 0.0321 |
| **Refine ls shift/su max** | 0.0009 |
| **Refine ls shift/su mean** | 0.0003 |
| **Refine ls structure factor coef** | F |
| **Refine ls weighting details** | w = 1/(\s$^2$(F) + 0.0001F$^2$) |
| **Refine ls weighting scheme** | sigma |
| **Refine ls wR factor gt** | 0.0436 |
| **Refine ls wR factor ref** | 0.0436 |

**Table S5**. Atomic coordinates and ADPs at 180 K.

| | **Wyckoff** | **x** | **y** | **z** | **Beq** | **Occ** |
|---|---|---|---|---|---|---|
| **Bi** | 3a | 0 | 0 | 0 | 0.0203(3) | 1 |
| **K** | 3b | 0.333333 | 0.666667 | 0.166667 | 0.0271(14) | 1 |
| **Cl** | 36i | 0.1084(6) | 0.3299(6) | 0.0698(2) | 0.0444(18) | 0.5 |
| **N** | 6c | 0.666667 | 0.333333 | 0.0502(9) | 0.043(5) | 1 |
| **C** | 6c | 0.666667 | 0.333333 | 0.1201(13) | 0.089(12) | 1 |
| | U11 | U22 | U33 | U12 | U13 | U23 |
| **Bi** | 0.0204(4) | 0.0204(4) | 0.0200(5) | 0.01019(18) | 0 | 0 |
| **K** | 0.0324(18) | 0.0324(18) | 0.016(2) | 0.0162(9) | 0 | 0 |
| **Cl** | 0.054(3) | 0.0316(19) | 0.041(2) | 0.0160(16) | −0.0051(17) | −0.0131(16) |

| | | | | | | |
|---|---|---|---|---|---|---|
| **N** | 0.044(6) | 0.044(6) | 0.041(10) | 0.022(3) | 0 | 0 |
| **C** | 0.116(17) | 0.116(17) | 0.035(15) | 0.058(9) | 0 | 0 |
